# The power of DNA based methods in probiotic authentication

**DOI:** 10.3389/fmicb.2023.1158440

**Published:** 2023-04-17

**Authors:** Hanan R. Shehata, Steven G. Newmaster

**Affiliations:** ^1^Natural Health Product Research Alliance, Department of Integrative Biology, College of Biological Science, University of Guelph, Guelph, ON, Canada; ^2^Department of Microbiology, Faculty of Pharmacy, Mansoura University, Mansoura, Egypt

**Keywords:** probiotic, authentication, shotgun metagenomic sequencing, amplicon-based high throughput sequencing, strain-specific, species-specific

## Abstract

**Introduction:**

The global probiotic market is growing rapidly, and strict quality control measures are required to ensure probiotic product efficacy and safety. Quality assurance of probiotic products involve confirming the presence of specific probiotic strains, determining the viable cell counts, and confirming the absence of contaminant strains. Third-party evaluation of probiotic quality and label accuracy is recommended for probiotic manufacturers. Following this recommendation, multiple batches of a top selling multi-strain probiotic product were evaluated for label accuracy.

**Methods:**

A total of 55 samples (five multi-strain finished products and 50 single-strain raw ingredients) containing a total of 100 probiotic strains were evaluated using a combination of molecular methods including targeted PCR, non-targeted amplicon-based High Throughput Sequencing (HTS), and non-targeted Shotgun Metagenomic Sequencing (SMS).

**Results:**

Targeted testing using species-specific or strain-specific PCR methods confirmed the identity of all strains/species. While 40 strains were identified to strain level, 60 strains were identified to species level only due to lack of strain-specific identification methods. In amplicon based HTS, two variable regions of 16S rRNA gene were targeted. Based on V5–V8 region data, ~99% of total reads per sample corresponded to target species, and no undeclared species were detected. Based on V3–V4 region data, ~95%–97% of total reads per sample corresponded to target species, while ~2%–3% of reads matched undeclared species (*Proteus* species), however, attempts to culture *Proteus* confirmed that all batches were free from viable *Proteus* species. Reads from SMS assembled to the genomes of all 10 target strains in all five batches of the finished product.

**Discussion:**

While targeted methods enable quick and accurate identification of target taxa in probiotic products, non-targeted methods enable the identification of all species in a product including undeclared species, with the caveats of complexity, high cost, and long time to result.

## Introduction

Probiotics are defined as “live microorganisms that, when administered in adequate amounts, confer a health benefit on the host” (Hill et al., 2014). The global probiotic market is growing rapidly, valued at ~USD 58.17 billion in 2021 (Grand-View-Research-Inc, 2022). To ensure probiotic product efficacy and safety, quality control measures are required. Among many aspects to be considered are authenticating product content of probiotic strains, which includes confirming strain identity, determining viable counts and confirming absence of contaminant strains (Patro et al., 2016). In addition to internal quality control measures, it is recommended that probiotic manufacturers undergo third-party evaluation of probiotic quality and label accuracy (Jackson et al., 2019), and communicating this third-party evaluation to consumers will help consumers identify high quality products (Jackson et al., 2019).

A critical point while performing internal quality control measures or third-party evaluation is choosing the test methods, which should be reliable, accurate, sensitive, and preferably fast and simple. The most commonly used methods for probiotic identification are DNA based methods. Several DNA based methods are used for probiotic strain or species identification including targeted methods (e.g., species-specific and strain-specific PCR) and non-targeted methods (e.g., High-Throughput Sequencing, HTS; Fasoli et al., 2003; Theunissen et al., 2005; Marcobal et al., 2008; Drago et al., 2010; Simmons et al., 2015; Morovic et al., 2016; Patro et al., 2016; Chen et al., 2017; Shehata et al., 2019). Targeted DNA based methods target a specific species or strain, while non-targeted methods can identify all strains/species in a product, which enable the detection of undeclared or contaminant species/strains (Morovic et al., 2016; Patro et al., 2016; Shehata and Newmaster, 2020a). Several targeted DNA based methods (species-specific or strain-specific) were developed for probiotic identification including conventional PCR and real-time PCR methods (Solano-Aguilar et al., 2008; Ahlroos and Tynkkynen, 2009; Achilleos and Berthier, 2013; Herbel et al., 2013; USP, 2015; Morovic et al., 2016; Shehata and Newmaster, 2020c; Shehata et al., 2021a,b, 2023).

The availability of probiotic identification methods has enabled several studies to investigate the authenticity of labeled probiotic species/strains found within several probiotic products of which some have reported incidences of non-compliance (Kolaček et al., 2017; Shehata and Newmaster, 2020a). Such incidences of non-compliance compromise consumer trust in the quality of probiotic products (Jackson et al., 2019). In this study, following the recommendation for third-party evaluation of probiotic product quality (Jackson et al., 2019), multiple batches of a top selling multi-strain probiotics, along with multiple batches of its raw ingredients were evaluated for label accuracy using a variety of molecular methods including targeted PCR, non-targeted amplicon-based HTS, and non-targeted shotgun metagenomic sequencing (SMS). The study demonstrates the strengths and weaknesses of the different authentication methods. To the best of our knowledge, this is the first study to evaluate and compare the performance of these DNA based methods in probiotic authentication for quality assurance.

## Materials and methods

### Sample collection and processing

A total of 55 probiotic samples were used in this study. The samples included five batches of a top selling multi-strain probiotic product in its finished product form (capsules containing a blend of 10 probiotic strains), and five batches from each of its 10 strains in the form of lyophilized powders. The total number of strains in all 55 samples was 100 strains (Supplementary Table S1). DNA was extracted from all samples using NucleoSpin Food kit (740945.50, Macherey Nagel, Germany), according to manufacturer’s instructions. DNA was eluted in 50 μL elution buffer. DNA samples were quantified using Qubit 4.0 Fluorometer, normalized to 1 ng/μL and were stored in a −20°C freezer until use.

### Species/strain content verification in probiotic samples

Targeted and non-targeted methods were used to verify the species/strain content in each of the samples, including species-specific or strain-specific PCR methods, 16S rRNA amplicon-based high-throughput sequencing (HTS), and Shotgun Metagenomic Sequencing (SMS).

### Targeted species-specific or strain-specific PCR for species/strain verification

A total of 55 samples were tested to verify the presence of a total of 100 strains using species-specific or strain-specific methods (Supplementary Tables S1, S2). Samples of *Lactobacillus acidophilus* La-14 (*n* = 10) and *Lacticaseibacillus paracasei* Lpc-37 (*n* = 10) were tested using strain-specific conventional PCR methods (USP, 2015). Samples of *Bifidobacterium animalis* subsp. *lactis* Bl-04 (*n* = 10) and *Lacticaseibacillus paraca*sei 8,700:2 (*n* = 10) were tested using strain-specific real-time PCR methods (Hansen et al., 2018; Shehata et al., 2023). Samples of *Lacticaseibacillus casei* Lc-11 (*n* = 10), *Ligilactobacillus salivarius* Ls-33 (*n* = 10), *Levilactobacillus brevis* Lbr-35 (*n* = 10), *Lactobacillus delbrueckii* subsp. *bulgaricus* Lb-87 (*n* = 10), *Lactiplantibacillus plantarum* HEAL9 and/or *Lactiplantibacillus plantarum* 299v (*n* = 15) were tested using species-specific primers (Morovic et al., 2016). Successful PCR amplification was verified by examining the PCR products on pre-cast 2% E-gel stained with SYBR Safe (G720802, Invitrogen).

### Non-targeted 16S rRNA amplicon based HTS

Sequencing libraries were prepared from five multi-strain finished products following the protocols in the 16S Metagenomic Sequencing Library Preparation Guide (Illumina, 2013). Both the V3–V4 (Illumina, 2013) and the V5–V8 regions (O’Callaghan et al., 2019) of the 16S rRNA gene were used. First stage PCR primers consisted of locus-specific sequences and overhang nucleotide sequences (Illumina, 2013; O’Callaghan et al., 2019). Each PCR reaction mix (25 μl total volume) contained 1× KAPA HiFi HotStart Ready Mix (KAPA Biosystems, United States), 0.2 μM of each primer (Integrated DNA Technologies, IDT, United States), and 5 μL of DNA. Thermal cycling conditions were 95°C for 3 min, 25 cycles of 95°C for 30 s, 55°C for 30 s and 72°C for 30 s, followed by 72°C for 5 min using a GeneAmp PCR System 9700 thermal cycler (Life Technologies, United States). PCR products were purified using AMPure XP beads (Beckman Coulter Genomics, United States). The purified PCR products were re-amplified using the Nextera XT Index Kit (Illumina, United States) to add indices and sequencing adaptors. The second stage PCR reaction mix (50 μL total volume) contained 1× KAPA HiFi HotStart Ready Mix, 5 μL each of Nextera™ XT Index Primers (Illumina), and 5 μL of purified PCR products from first stage PCR. PCR thermal cycling conditions were 95°C for 3 min, 8 cycles of 95°C for 30 s, 55°C for 30 s and 72°C for 30 s, followed by 72°C for 5 min using a GeneAmp PCR System 9700 thermal cycler. PCR products were purified using AMPure XP beads.

### Non-targeted shotgun metagenomic sequencing

Sequencing libraries were prepared from the five multi-strain finished products using Nextera™ Flex DNA Library Prep kit following the instructions in the Nextera™ DNA Flex Prep Reference Guide (Illumina, 2019). Genomic DNA (~300 ng) was fragmented using Nextera transposome and tagged with adapter sequences. Index adapters and sequences required for sequence cluster generation were added to the adapter-tagged DNA using a limited-cycle PCR program. PCR thermal cycling conditions were: 72°C for 3 min and then 95°C for 30 s; 5 cycles of 95°C for 10 s, 55°C for 30 s and 72°C for 30 s, followed by 72°C for 5 min followed by a hold at 10°C using a GeneAmp PCR System 9700 thermal cycler. The amplified libraries were then purified using sample purification beads.

### DNA sequencing using MiSeq system

The quality and quantity of both the amplicon-based sequencing libraries and the shotgun metagenomic sequencing libraries were assessed by Fragment Analyzer Automated CE System using the dsDNA 935 Reagent Kit (Agilent Technologies). DNA was also quantified using a Qubit® Fluorometer and a Qubit® dsDNA BR Assay Kit (Thermo Fisher Scientific). The purified libraries were normalized and pooled in equal molar ratios based on their DNA concentrations. The pooled libraries were denatured with NaOH and diluted with hybridization buffer before sequencing. PhiX (Illumina) at 1.5% was included as an internal control. Sequencing was conducted using a MiSeq sequencer with a MiSeq 3 reagent kit (Illumina) and 2 × 300 paired-end cycles according to the manufacturer’s protocol. 16S rRNA amplicon based HTS and SMS were conducted at the Laboratory Services Division, University of Guelph.

### HTS and SMS data analysis

The MiSeq Sequencer System Software (Illumina) was used to filter raw sequence reads and remove low quality sequences. Adapter sequences were trimmed, and sequences were further filtered to remove short sequences using the fast QC APP in BaseSpace (Illumina). Sequences that passed the filter criteria (length of 280 bp with averaged quality score of 30) were used for further analysis.

For amplicon based HTS data analysis, the 16S Metagenomics analysis pipeline in BaseSpace (Illumina) was used to make taxonomic assignments and generate data summaries of the proportions of taxa present. The sequence database for the 16S rRNA gene target was Greengenes V13_5 (DeSantis et al., 2006). For SMS data analysis, the sequences were then assembled to each of the reference genome sequences using Geneious software v10.2.6 (Biomatters Ltd) to generate contigs. Additionally, shotgun data were analyzed using APD (Advanced Probiotics species Detection; Seol et al., 2019).

## Results

### Targeted species-specific or strain-specific PCR for species/strain verification

**Figure 1:**
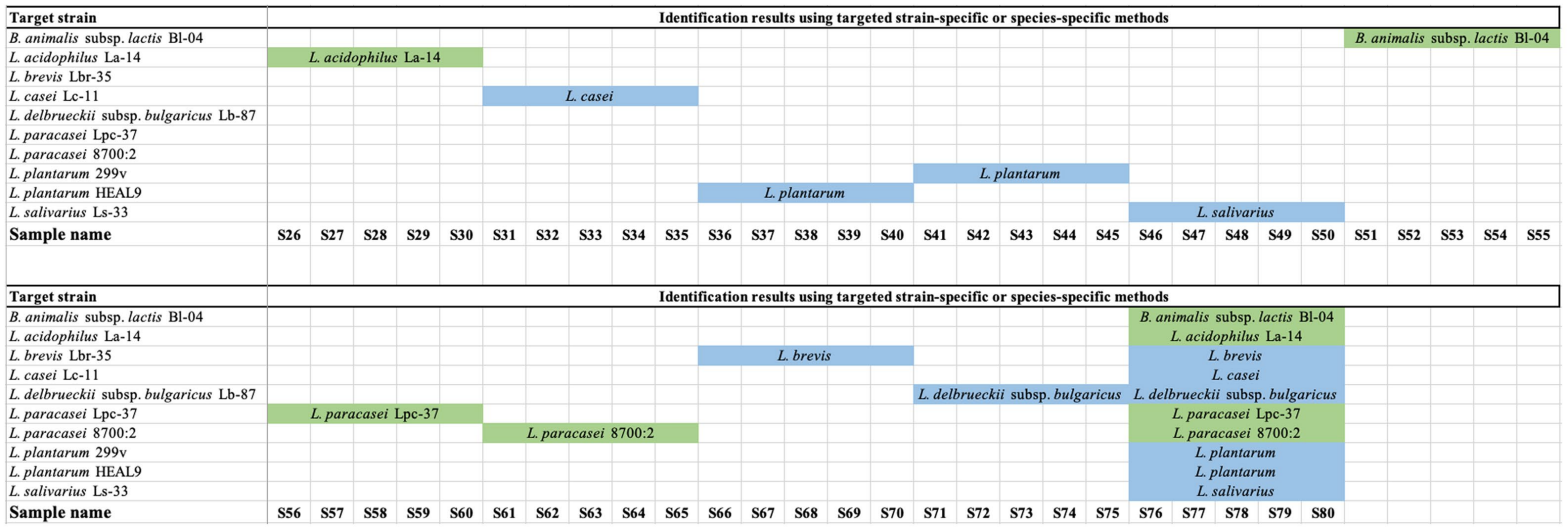
Results of targeted species-specific or strain-specific PCR testing of probiotic samples. Green indicates a declared strain was verified to strain level. Blue indicates a declared strain was verified to species level.

A total of 100 strains were tested using species-specific or strain-specific PCR assays (USP, 2015; Morovic et al., 2016; Hansen et al., 2018; Shehata et al., 2023). All samples of *Lactobacillus acidophilus* La-14 (*n* = 10), *Bifidobacterium animalis* subsp. *lactis* Bl-04 (*n* = 10), *Lacticaseibacillus paracasei* 8,700:2 (*n* = 10), and *Lacticaseibacillus paracasei* Lpc-37 (*n* = 10) were tested using strain-specific PCR assays and were identified as the correct target strains (Figure 1; Supplementary Table S1). Samples of *Lacticaseibacillus casei* Lc-11 (*n* = 10), *Ligilactobacillus salivarius* Ls-33 (*n* = 10), *Levilactobacillus brevis* Lbr-35 (*n* = 10), *Lactobacillus delbrueckii* subsp. *bulgaricus* Lb-87 (*n* = 10), and *Lactiplantibacillus plantarum* HEAL9 and/or *Lactiplantibacillus plantarum* 299v (*n* = 15) were tested using species-specific PCR assays and were identified as the correct target species (Figure 1; Supplementary Table S1). The presence of two distinct *L. plantarum* strains in the five multi-strain samples could not be confirmed due to the lack of strain-specific assays for *L. plantarum* HEAL9 and *L. plantarum* 299v.

### Non-targeted 16S rRNA amplicon based HTS

**Figure 2:**
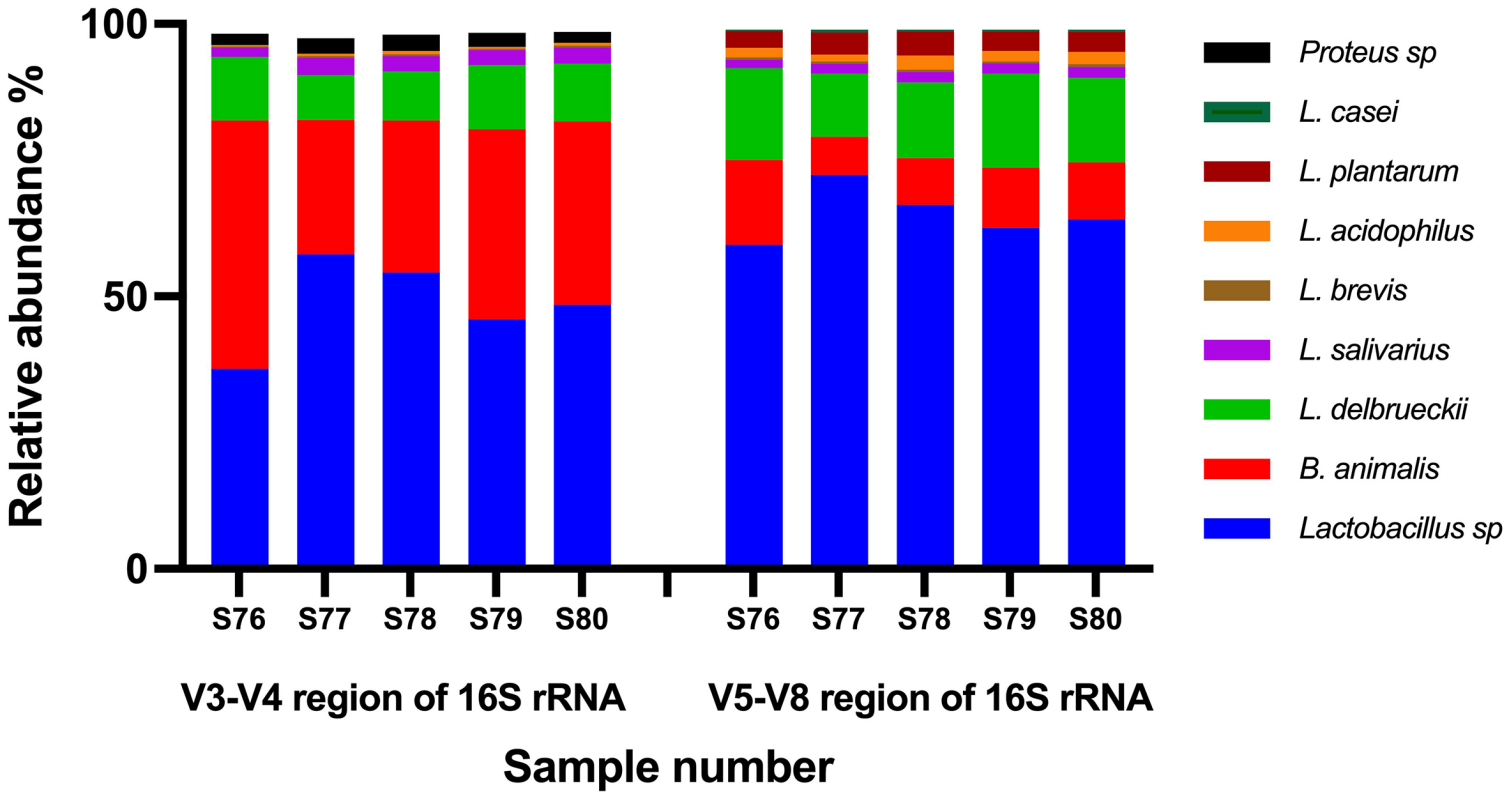
Results of non-targeted testing of probiotic samples using amplicon-based High Throughput Sequencing. Two variable regions of 16S rRNA gene were targeted (V3–V4 and V5–V8). Shown is the percentage relative abundance of the Operational Taxonomic Units identified in each sample.

Two variable regions of 16S rRNA gene were targeted (V3–V4 and V5–V8). The number of reads per sample ranged from 206,878 to 253,030 for V3–V4 region and ranged from 167,001 to 207,399 for V5–V8 region. For data de-noising, Operational Taxonomic Units (OTUs) represented by less than 0.2% of total reads per sample were excluded. Based on V5–V8 region data, ~99% of total reads per sample corresponded to target species (Figure 2), and no undeclared species were detected. Based on V3–V4 region data, ~95%–97% of total reads per sample corresponded to target species, while ~2%–3% of reads matched undeclared species (*Proteus mirabilis* and *Proteus myxofaciens*; Figure 2). To test whether *Proteus* species were truly present as contaminants in the products, all five batches were plated on MacConkey agar, a medium that is selective for Gram-negative and enteric bacteria. No colonies were retrieved on MacConkey agar following incubation at 37°C for 48 and 72 h, indicating that all batches were free from viable *Proteus* species.

### Non-targeted shotgun metagenomic sequencing

Five batches of the finished product were tested for their strain content using SMS. The total number of reads that resulted from SMS ranged from 12.9 to 18.4 million reads per sample, and the total number of reads that passed the quality filtration criteria ranged from 11.6 to 16.4 million reads per sample. Sequencing reads assembled to all the 10 target genomes declared on the product label in all five batches (Supplementary Table S3). The total number of reads that assembled to all 10 target genomes exceeded the total number of reads in each sample, which indicates that a number of reads assembled to multiple target genomes. When analyzed using a coverage-based pipeline, APD (Seol et al., 2019), SMS data showed that all eight species of the 10 strains declared on the test product labels were detected in all five lots, and no undeclared probiotic species were detected using this pipeline (Supplementary Table S4).

## Discussion

The strain content in an authentic and high quality probiotic product should match what is declared on its label, including identity and quantity of probiotic strains (Fusco et al., 2022). In terms of identity, probiotic strains should be identified using scientifically valid genus and species names, as well as strain names (FAO/WHO, 2002). In terms of quantity, viable cell counts should meet declared viable counts, at the end of shelf life (Tripathi and Giri, 2014; Kolaček et al., 2017; Sánchez et al., 2017; Fusco et al., 2022). In addition to strain identity and quantity, the serving size that delivers an effective dose of probiotics, recommended storage conditions, health claims that are supported by scientific evidence, and expiration dates should be declared on product labels (Council-for-Responsible-Nutrition-and-International-Probiotics-Association, 2017).

Mislabeling in commercial probiotic products is frequently reported worldwide with products not meeting label claims of probiotic strains (missing strains, presence of undeclared strains, strain substitution, or use of scientifically invalid nomenclature) or not meeting the declared viable counts before expiration date (Kolaček et al., 2017; Shehata and Newmaster, 2020b; Fusco et al., 2022). Issues with species or strain identity can largely be attributed to misidentification due to lack of proper identification methods (Kolaček et al., 2017). An example is labeling *Bifidobacterium longum* subsp. *longum* as *B. longum* subsp. *infantis* (Lewis et al., 2016; Patro et al., 2016; Shehata and Newmaster, 2020a). Hence, reliable identification methods are essential for high quality probiotic products. The most commonly used methods for probiotic identification are DNA based methods. DNA based methods detect DNA only and hence should be coupled with other culture-dependent or culture-independent methods to confirm cell viability, but this is beyond the scope of this study. DNA based methods include targeted methods (species-specific and strain-specific PCR methods) and non-targeted methods (e.g., high-throughput sequencing, HTS; Fasoli et al., 2003; Theunissen et al., 2005; Marcobal et al., 2008; Drago et al., 2010; Simmons et al., 2015; Morovic et al., 2016; Patro et al., 2016; Chen et al., 2017; Shehata et al., 2019; Shehata and Newmaster, 2020c; Shehata et al., 2021b). In this study, an array of molecular methods including targeted PCR, non-targeted amplicon based HTS, and non-targeted shotgun metagenomic sequencing were used to conduct third-party evaluation of label accuracy in multiple batches of a top selling multi-strain probiotic product, along with its raw ingredients.

Targeted strain-specific PCR methods are available for *L. acidophilus* La-14, *Bifidobacterium animalis* subsp. *lactis* Bl-04, *L. paracasei* 8,700:2 and *L. paracasei* Lpc-37. Thus, samples of the four strains were identified to the strain level, while only species-specific PCR methods were available for *L. casei* Lc-11, *L. salivarius* Ls-33, *L. brevis* Lbr-35, *L. delbrueckii* subsp. *bulgaricus* Lb-87, *L. plantarum* HEAL9 and *L. plantarum* 299v, thus, samples belonging to the six strains were identified to species level only (Figure 1; Supplementary Table S1). Using species-specific primers, the presence of two distinct strains of *L. plantarum* could not be verified in finished products due to lack of strain specific assays for these two closely related strains (Håkansson et al., 2019). On the other hand, availability of strain-specific assays for *L. paracasei* 8,700:2 and *L. paracasei* Lpc-37 enabled the identification of two distinct strains of *L. paracasei* in finished products. Such strain-specific methods make it possible to authenticate products as raw ingredients pre-blending as well as post-blending. Overall, 40 out of 100 target strains were identified to the strain level, while 60 targets were identified to species level only.

The use of non-targeted amplicon-based HTS of V3–V4 region of 16S rRNA gene revealed that ~95%–97% of total reads per sample corresponded to target species while amplicon-based HTS of V5–V8 region of 16S rRNA gene revealed that ~99% of total reads per sample corresponded to target species. Based on V5–V8 region data, no undeclared species were detected above the threshold of 0.2% of total reads per sample. Based on V3–V4 region data, ~2%–3% of reads matched *Proteus mirabilis* and *Proteus myxofaciens*. Attempts to retrieve culturable *Proteus* species failed, confirming the absence of viable *Proteus* species in any of the products. The presence of a low read count of *Proteus* in the V3–V4 region data only and absence of reads identified as *Proteus* in the V5–V8 region data as well as failure to retrieve culturable *Proteus* may indicate an artifact from erroneous sequences resulting from PCR or sequencing errors (Pfeiffer et al., 2018). Another possibility is that this trace amount of *Proteus* DNA may have originated from one of the excipients in the finished products, as the genus *Proteus* is widely distributed in nature and can be isolated from soil including agricultural soil (Armbruster and Mobley, 2012).

In this study, sequencing data denoising was conducted by excluding OTUs represented by less than 0.2% of total reads per sample. HTS data typically undergoes data filtration based on quality, read length, and ambiguous base calls. Further de-noising of HTS sequencing data is recommended (Kunin et al., 2010; Zaura, 2012), with multiple proposed methods for denoising (Laehnemann et al., 2015), although it may result in false negative results for low abundance taxa (Zaura, 2012).

The use of 16S rRNA HTS has become a very popular approach to characterize the taxonomic composition of complex bacterial communities (Darwish et al., 2021). The 16S rRNA gene in bacteria contains nine hypervariable regions (V1 to V9), flanked by highly conserved regions (Yarza et al., 2014). Full length 16S rRNA is ~1,500 bp, which is longer than the read length possible from current high throughput sequencing platforms (Alcon-Giner et al., 2017). Thus, a variable region of 16S rRNA is typically selected for high throughput sequencing. The selection of variable regions of 16S rRNA gene to characterize bacterial communities is important because of the preferential primer binding to the different taxa (O’Callaghan et al., 2019). Thus, sequencing different variable regions can result in different profiles of bacterial communities (Alcon-Giner et al., 2017). The different variable regions can also have variable taxonomic discriminatory powers. Poor taxonomic resolution is expected in amplicon-based HTS due to the short read length (Zaura, 2012), and can result in identification to genus level only (Jackson et al., 2019). This can be problematic when dealing with probiotic products which can contain 10 or more species of the genus *Lactobacillus* and the reclassified *Lactobacilli* genera in the same product. Although, the genus *Lactobacillus* has been reclassified into 25 genera (Zheng et al., 2020), the Greengenes 16S rRNA database, that was used for OTU assignment, is no longer being updated and still uses the old taxonomy (Qiao et al., 2022). In this study, two variable regions were used, V3–V4 and V5–V8. The V3–V4 region is commonly used for bacterial community characterization from various sources (Almeida et al., 2019; Amrane et al., 2019; Chen et al., 2020; Shehata et al., 2020; Shehata and Newmaster, 2020a). The V5–V8 region of 16S rRNA gene was found to have sufficient sequence variation to allow accurate species level identification of the genus *Lactobacillus* (O’Callaghan et al., 2019). The use of the V5–V8 region of 16S rRNA gene in this study resulted in a higher resolution compared to V3–V4 region, where most species were identified to species level (Figure 2).

In this study, we also used shotgun metagenomic analysis, which is significantly more expensive and requires higher input of DNA but also known to introduce less PCR bias and artifacts compared to amplicon-based HTS (Alcon-Giner et al., 2017). SMS was also found to identify a significantly higher number of bacterial species compared to amplicon-based HTS, which is important when analyzing complex microbial communities (Ranjan et al., 2016). Using shotgun metagenomic analysis, sequencing reads assembled to the genomes of all 10 strains declared on the product label in all five batches. A number of reads assembled to multiple target genomes, which can be attributed to the high sequence similarity between the target strains, with 2 *L. plantarum* and 2 *L. paracasei* strains in the same product. This hindered further quantitative analysis of SMS data to find relative abundance of the strains in the products (Supplementary Table S3).

## Conclusion

Using multiple DNA based methods, the tested probiotic samples were verified to contain the declared species/strains in all tested batches, indicating a consistently high-quality product. The lack of strain-specific identification methods for six of the target strains hindered the strain-level identification. With species level identification, it was not possible to confirm the presence of two different strains of *L. plantarum*. Comparing the performance of targeted and non-targeted methods showed that while targeted methods can quickly and accurately confirm the presence or absence of target taxa in a product with high taxonomic resolution, high sensitivity and at low cost, non-targeted methods can identify species present in a product including undeclared species and contaminant species, but this technique is more expensive, more complex, time-consuming, and may have poor taxonomic resolution, especially with amplicon-based HTS. The need for reliable strain-specific identification methods is growing with the increasing number of probiotic strains and the expanding probiotic market size.

## Data availability statement

The datasets presented in this study can be found in online repositories. The names of the repository/repositories and accession number(s) can be found at: Sequence Read Archive in NCBI under accession number PRJNA946761.

## Author contributions

HS carried out the experiments, helped to analyze the data, and wrote the manuscript. SN helped to design the study, facilitated sample acquisition, and edited the manuscript. All authors contributed to the article and approved the submitted version.

## Funding

This study was supported by the Natural Health Product Research Alliance (NHPRA), University of Guelph.

## Conflict of interest

The authors declare that the research was conducted in the absence of any commercial or financial relationships that could be construed as a potential conflict of interest.

## Publisher’s note

All claims expressed in this article are solely those of the authors and do not necessarily represent those of their affiliated organizations, or those of the publisher, the editors and the reviewers. Any product that may be evaluated in this article, or claim that may be made by its manufacturer, is not guaranteed or endorsed by the publisher.
