# Supplementary material for: The power of DNA based methods in probiotic authentication
Source: Front Microbiol. 2023 Apr 17;14:1158440. doi: 10.3389/fmicb.2023.1158440 (PMC10150049; doi:10.3389/fmicb.2023.1158440)
Supplement: Supplementary file 2 [file Table_2.DOCX]

**Table S2: Primer and probe sequences used in targeted species-specific or strain-specific PCR**

| **Target species or strain** | **Primer or probe sequence  (5′–3′)** | **Product size** | **Reference** |
| --- | --- | --- | --- |
| *Lactiplantibacillus plantarum* | F: AAATCGGCATCTTTGTATTCATTTTGGGCA | 124 bp | (Morovic et al., 2016) |
|  | R: TTGGTGGTCGTTTCACTGTTTTGACA |  |  |
|  |  |  |  |
| *Levilactobacillus brevis* | F: CCTTTGATCGTTCCAAACTGACACCG | 250 bp |  |
|  | R: GCACCCAGATAAGATCCCCCG |  |  |
|  |  |  |  |
| *Lactobacillus delbruekii* subsp. *bulgaricus* | F: GGATCGGCGGCTCATACTTG | 502 bp |  |
|  | R: GGTGAGTCCTTGCTTACGTCAGG |  |  |
|  |  |  |  |
| *Lacticaseibacillus casei* | F: AGCTGGACAAGTTCGTTCATGCC | 200 bp |  |
|  | R: GAAGACCTGTGAGTTGCCCTGAATC |  |  |
|  |  |  |  |
| *Ligilactobacillus salivarius* | F: TATTGACCAAGTGAGTGCAAGTCTGTTGA | 343 bp |  |
|  | R: ATGATGTTGGTGGTCGTTTCTCAGTACTTA |  |  |
|  |  |  |  |
| *Lactobacillus acidophilus* La-14 | F: AAACTGCAATTTAAGATTATGAGTTTC | 184 bp | (USP, 2015) |
|  | R: GGTACCGTCTTGATTATTAGTGTA |  |  |
|  |  |  |  |
| *Lacticaseibacillus paracasei* Lpc-37 | F: GTTTGTGGCGGCGTAACTTC | 273 bp |  |
|  | R: GGTGATCCTGAACGCGGTT |  |  |
|  |  |  |  |
| *Lacticaseibacillus paracasei* 8700:2 | F: GGAACTCGTAGCATCTACTAAGC | 149 bp | (Shehata et al., 2023) |
|  | R: CTATGGCCTTGTCTCCTTCTTC |  |  |
|  | P: AGAATCCACAAGAGACGCCCAAAGT  (56-FAM and ZEN – 3IABkFQ) |  |  |
|  |  |  |  |
| *Bifidobacterium animalis* subsp. *lactis* Bl-04 | F: CTTCCCAGAAGGCCGGGT | 98 bp | Modified from (Hansen et al., 2018) |
|  | R: CGAGGCCACGGTGCTCATATAGA |  |  |
|  | P: CGAAGATGATGTCGGAACACAAACACCCGG  (56-FAM and ZEN – 3IABkFQ) |  |  |
